# Supplementary material for: Splice-Junction-Based Mapping of Alternative Isoforms in the Human Proteome
Source: Cell Rep. Author manuscript; Available in PMC 2020 Jan 15. (PMC6961840; doi:10.1016/j.celrep.2019.11.026)
Supplement: 2 [file NIHMS1546469-supplement-2.zip › DF1/PXD010154/AdrenalGland/AdrenalGland_14_PPP1R12A_ISEMEEELKR.pdf]

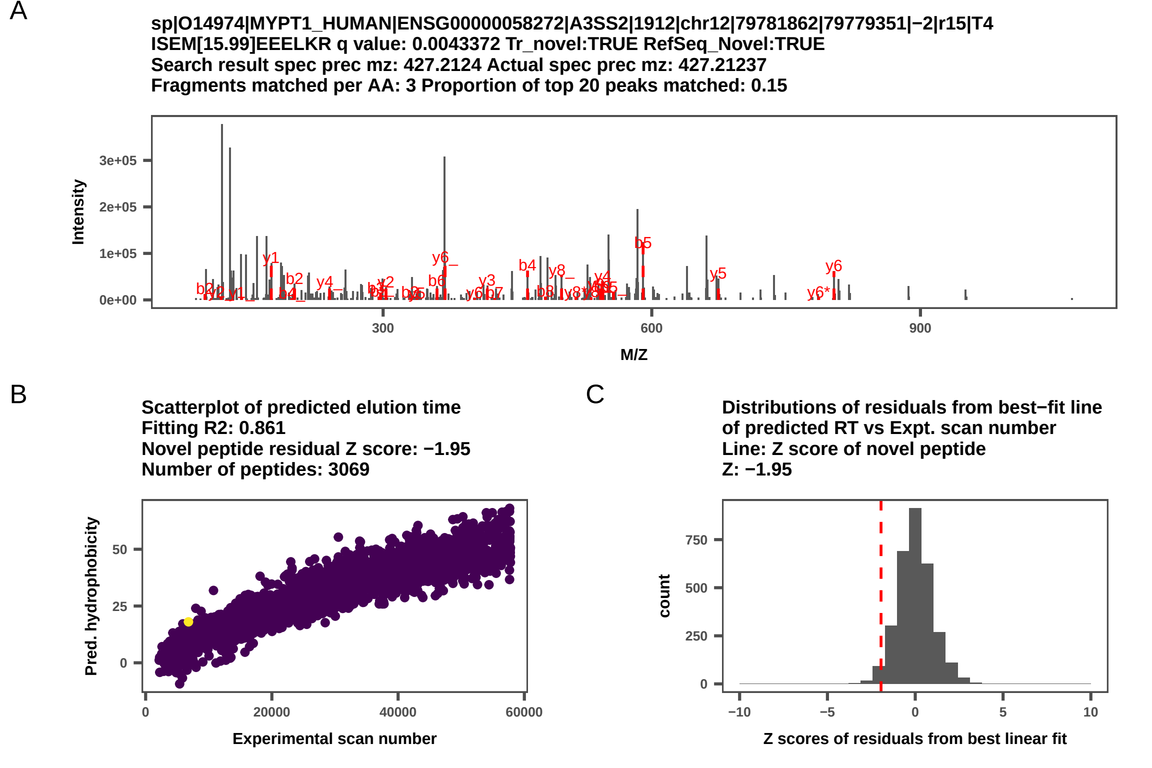
A
sp|O14974|MYPT1_HUMAN|ENSG00000058272|A3SS2|1912|chr12|79781862|79779351|−2|r15|T4
ISEM[15.99]EEELKR q value: 0.0043372 Tr_novel:TRUE RefSeq_Novel:TRUE
Search result spec prec mz: 427.2124 Actual spec prec mz: 427.21237
Fragments matched per AA: 3 Proportion of top 20 peaks matched: 0.15
Intensity
3e+05
2e+05
1e+05
0e+00
300
600
900
M/Z
b5
y6
y6_
b4
y8_
y1
y5
b6
y3
b2
y4_
y4
y6*
B
Scatterplot of predicted elution time
Fitting R2: 0.861
Novel peptide residual Z score: −1.95
Number of peptides: 3069
Pred. hydrophobicity
50
25
0
0
20000
40000
60000
Experimental scan number
C
Distributions of residuals from best−fit line
of predicted RT vs Expt. scan number
Line: Z score of novel peptide
Z: −1.95
count
750
500
250
0
−10
−5
0
5
10
Z scores of residuals from best linear fit
